# Interaction of mindfulness disposition and instructional self-talk on motor performance: a laboratory exploration

**DOI:** 10.7717/peerj.7034

**Published:** 2019-06-06

**Authors:** Yi-Hsiang Chiu, Frank J.H. Lu, Diane L. Gill, Tzu-Wen Lin, Chiu-Chen Chang, Shu-Ching Wu

**Affiliations:** 1 Department of Physical Education, Chinese Culture University, Taipei, Taiwan; 2 Department of Kinesiology, University of North Carolina at Greensboro, Greensboro, NC, United States of America; 3 Department of Physical Education, National Taiwan Normal University, Taipei, Taiwan; 4 Department of Kinesiology, Health, and Leisure Studies, National University of Kaohsiung, Kaohsiung, Taiwan; 5 Center for General Education, Ling-Tung University, Taichung, Taiwan

**Keywords:** Attention disposition, Cognitive strategies, Motor skill performance, Thought control

## Abstract

In considering that high mindfulness disposition individuals possess a unique ability to maintain attention and awareness, and attention is one of the key mechanisms of instructional self-talk, the purpose of this study was to examine the interaction of mindfulness disposition and instructional self-talk on motor performance. Forty-nine college students (*M age* = 18.96 ± 1.08) with high/low mindfulness disposition (high *n* = 23; low *n* = 26) selected out of 126 college students performed a discrete motor task (standing long jump) and a continuous motor task (line tracking task) under instructional and unrelated self-talk conditions. Two separate 2 (self-talk type) X 2 (high/low mindfulness) mixed design ANOVA statistical analyses indicated that mindfulness disposition interacted with unrelated self-talk in the line tracking task. Specifically, low mindfulness participants performed poorer than high mindfulness participants in line tracking task under unrelated self-talk. Further, participants performed better in both standing long jump and line tracking under instructional self-talk than unrelated self-talk. Results not only revealed the triangular relationships among mindfulness, self-talk, and motor performance but also indirectly support the role of attention in self-talk effectiveness. Limitations, future research directions, and practical implications were discussed.

## Introduction

Self-talk is one of the most popular skills in psychological skills training (Gould et al., 1991, p. 121), and one that has been widely used by performers (Weinberg & Gould, 2015; Williams & Krane, 2015). Self-talk is so widely used by athletes and exercisers because research shows that self-talk can effectively enhance sport and motor performance (Edwards, Tod & McGuigan, 2008; Kolovelonis, Goudas & Dermitzaki, 2011; Dickens, Van Raalte & Hurlburt, 2018); Hardy, Comoutos & Hatzigeorgiadis, 2018; Hatzigeorgiadis et al., 2011; Weinberg, Miller & Horn, 2012. Also, the benefits of self-talk have been further supported by both narrative and systematic reviews (e.g.,  Hardy, 2006; Hatzigeorgiadis et al., 2011; Tod, Hardy & Oliver, 2011). According to Hatzigeorgiadis et al. (2011), the global effect size of self-talk on motor performance is positive and moderate (effect size, ES = 0.48) but ranges from 0.22 to 1.31. To deepen our knowledge of the effects of self-talk on motor performance, Tod, Hardy & Oliver (2011) suggested that researchers not only continue examining the “first-generation questions”, that is, the effects of self-talk on performance; but also explore the “second-generation questions”, that is, investigation of moderators and mediators underlying the self-talk-motor performance relationship, such as self-efficacy (Chang et al., 2014), self-confidence (Hatzigeorgiadis et al., 2009), and task characteristics (Hatzigeorgiadis et al., 2011). Hence, the aim of this study was to explore the effects of instructional self-talk on motor performance; specifically, the mechanism underlying self-talk-performance relationship.

Theoretically, self-talk is a cognitive strategy that individuals use to talk to themselves either silently or aloud to interpret lived perceptions, to change evaluations and beliefs, and to give instructions or reinforcements (Hardy, Gammage & Hall, 2001; Hardy, 2006). For example, a tennis player may talk to himself and say “…hit the ball to the left bottom corner…” or “…forget about the last point, now focus on the next point,” or “common on, you can it!” All these statements, either spoken loudly or silently, are good examples of self-talk. According to the extant literature, self-talk includes the thoughts that a performer expresses in language or statement, and uses implicitly or explicitly before, during or after the execution of an activity or a sports skill (Zetou, Nikolaos & Evaggelos, 2014). Zetou, Nikolaos & Evaggelos (2014) contended that the self-talk process usually happens unconsciously; it affects not only the performers’ feelings but also their actions.

Notably, self-talk can be of different types including positive, negative, motivational, and instructional self-talk. Among all types of self-talk, instructional self-talk is particularly worthy of further examination because students learning a motor skill are given many verbal instructions (McGill, 2004; pp. 247–258). Instructional self-talk occurs when task performers have to focus on technical, tactical, or kinesthetic components of motor tasks (Hardy, 2006; Hardy, Gammage & Hall, 2001; Hatzigeorgiadis, Theodorakis & Zourbanos, 2004). Therefore, when performers apply instructional self-talk they need to emphasize the cognitive components of motor tasks. By doing so, performers can direct or redirect their attention to the task-relevant cues when performing (Hardy, 2006; Hatzigeorgiadis, Theodorakis & Zourbanos, 2004; Landin, 1994; Nideffer, 1993; Theodorakis et al., 2000).

Empirical studies examining the effects of instructional self-talk on motor performance yielded mixed results. For example, Theodorakis et al. (2000) assigned 72 male soccer players to shoot at a goal from a distance of 15 yards. Results found the instructional group performed better than the control group. Similar results were also found in studies by using different motor tasks such as basketball passing accuracy and speed (Boroujeni & Shahbazi, 2011), softball throwing accuracy (Chang et al., 2014), handball accuracy (Zourbanos et al., 2013), and basketball free throw shooting accuracy (Abdoli et al., 2018). In contrast, some research found instructional self-talk has no effect on the motor task. For example, Zourbanos et al. (2013) assigned 40 primary school students to perform handball throwing accuracy with the dominant hand and found no difference between instructional self-talk and control group. Similar results were also found in Theodorakis et al.’s (2000) early research examining the effect of instructional self-talk on a 3-min endurance sit-up task. Recently, Hardy, Begley & Blanchfield (2015) compared the effects of motivational self-talk and instructional self-talk on Gaelic football shooting accuracy using the non-dominant foot. Results indicated there was no difference between the two types of self-talk on shooting accuracy in skilled athletes.

The mixed results of the effects of instructional self-talk on motor performance prompted researchers to propose factors underlying the relationship between instructional self-talk and motor performance. Specifically, the attentional process during instructional self-talk has been suggested by researchers. Theodorakis et al. (2000,  p.266) explained that the use of instructional self-talk involves attentional process; performers not only have to focus on the relevant cues in the environment but also have to be aware of the specific technical and mechanical aspects of the skills. Zourbanos et al. (2013, p. 929) proposed that when performers are young and have no experience on a motor task (e.g., novel skill) the attentional demands are very high. Zourbanos et al. (2013, p. 929) explained that by using instructional self-talk performers can focus on the key elements of the movement and dismiss irrelevant stimuli. Thus, it seems that attention might underlie the instructional self-talk and motor performance relationship.

Attention is “*the behavioral and* cognitive process * of selectively concentrating on a discrete aspect of information, whether deemed subjective or objective while ignoring other perceivable information*” (Anderson, 2004; p. 519). Attention is also referred to as a state of effort and arousal (Schmidt & Lee, 2011), and the cognitive ability “*to possess something in the mind consciously or unconsciously in a clear and vivid form of one out of what seem several simultaneous objects or trains of thought* (Anderson, 2004; p. 519)”. Motor behavior scholars suggest that attention allows performers to allocate limited cognitive resources and handle it effectively. By doing so, they can successfully achieve behavioral goals (Schmidt & Lee, 2011; 97–99).

In considering the importance of attention in motor performance, we propose that performers’ mindfulness disposition- a behavioral tendency to be attentive- might influence the relationship between instructional self-talk and motor performance. According to Brown & Ryan (2003, p. 822–823) mindfulness disposition refers to “...*tendency for having an open or receptive awareness and attention which is reflected in a more regular or sustained consciousness of ongoing events and experiences*.” Many studies support that high mindfulness individuals have high attention ability and perform better on motor tasks than low mindfulness performers (Djikic, Langer & Stapleton, 2008; Kee et al., 2012; Kee & Liu, 2011; Mills & Allen, 2000). Thus, when examining the effects of instructional self-talk on motor performance it is imperative to examine participants’ mindfulness disposition because their attention tendency might be a key variable affecting the instructional self-talk and motor performance relationship.

In addition, the triangular relationship among instructional self-talk, mindfulness disposition, and motor performance suggests several theoretical questions that previous research has never explored. First, is the role of mindfulness disposition on motor performance. Past research examining the effects mindfulness on motor performance can be classified as (a) does mindfulness training influence motor performance (e.g., Kee et al., 2012; Kee et al., 2013; Mills & Allen, 2000; Zhang et al., 2016), or (b) how does mindfulness disposition influence motor performance (e.g., Kee et al., 2012). Generally, research has found mindfulness induction enhances motor performance (e.g., Kee et al., 2012; Kee et al., 2013; Mills & Allen, 2000; Zhang et al., 2016), or high mindful disposition participants performed better than low mindful individuals (e.g., Kee et al., 2012). Despite these efforts, research examining the effects of self-talk on motor performance has not examined how mindfulness disposition influences different types of motor tasks.

According to Schmidt & Lee (2011), motor tasks can be classified as discrete, continuous, or serial; and, each type of motor task requires a different attentional process. A discrete task, such as basketball free throw or soccer kicking, which is characterized by a recognizable beginning and end, requires performers to pay attention to external stimuli (e.g., target or goal), and then to act. Generally, the movement time for discrete motor tasks is relatively short. For a continuous task, such as swimming, steering a car, or tracking, which is characterized by having no recognizable beginning and end, performers must pay and maintain attention over a certain period until the task is completed. By doing so, performers can complete the motor task correctly. A serial motor task is neither discrete nor continuous, but usually comprised of a series of individual movements tied together in time to make some discrete tasks “whole” such as playing the piano or performing a gymnastics routine. It requires performers to pay and maintain attention to both individual movement and the whole routine of the motor task. Thus, will those with different mindful dispositions perform differently on different types of motor tasks during self-talk needs further examined. Past research on the effects of instructional self-talk on motor performance generally focused on one type of motor task, such as softball throwing (e.g., Chang et al., 2014), soccer shooting (e.g., Theodorakis et al., 2000) or basketball passing (e.g., Boroujeni & Shahbazi, 2011), without comparing how self-talk influences different types of motor tasks. Thus, will instructional self-talk has the same effects on different motor tasks is another question to be examined.

Moreover, past research examining the self-talk and motor performance relationship generally designed a control group such as “unrelated self-talk” (e.g., Chang et al., 2014) or summary feedback (e.g., Theodorakis et al., 2000), to prevent participants from automatically using another type of self-talk (e.g., motivational, positive, or negative) that might contaminate the experimental effects. Under such experimental control, will unrelated self-talk interact with mindful disposition on different motor tasks is worthy of investigation.

Further, Lewin (1953) proposed an interactionist approach of personality suggests that human behavior is the function of person and environment. *B* = *f*(*P*, *E*). Thus, when researchers examining the effects of self-talk on motor performance, they must consider that mindfulness disposition might interact with self-talks on different type of motor tasks. Using the aforementioned triangular relationships among mindfulness disposition, self-talk and motor tasks as an example. For high mindful individuals, they may perform well on motor task that requires the high ability of attention (e.g., continuous motor task) even under unrelated self-talk. In contrast, for low mindfulness individuals, they might perform poorly in the motor task that requires high attention under unrelated self-talk but remains unaffected for motor task that requires a short time of attention (e.g., discrete motor task). Therefore, to examine the interaction of mindfulness disposition and self-talk on different motor tasks can be a starting point to solve the puzzle of why instructional self-talk cannot lead to positive effects for motor performance.

Built on the above rationale, the purpose of the present study was to examine the interactive effects of mindfulness disposition and self-talk on motor performance. We proposed the following hypotheses: (i) Mindfulness disposition would interact with self-talk in standing long jump; (ii) Mindfulness disposition would interact with self-talk in line tracking.

## Method

### Phase 1: selection of participants

First, we recruited participants in PE classes at a university. We informed participants that the purpose of the study was to explore college students’ general psychological disposition and motor skills. Participants who were interested in this study signed a consent form and completed a demographic questionnaire and a Chinese version of the mindful attention awareness scale (MAAS). The MAAS (Brown & Ryan, 2003) consists of 15 items for assessing mindfulness disposition. The items are comprised of statements about everyday experiences for gauging frequency of receptive awareness of and attention to present-moment events and experiences. A sample question is “... I rush through activities without being really attentive to them.” The 6-point scale, ranging from 1 (almost always) to 6 (almost never), was used. Higher mean scores derived from all 15 items correspond to a high mindfulness disposition. Previous work suggests that MAAS is a single factor scale and has acceptable validity and reliability (alpha coefficients above .80, Brown & Ryan, 2003). The alpha coefficient of the scale for the current sample was .90. It took about 15 min to complete a consent form, demographic questionnaire, and MMAS.

A total of 126 participants voluntarily completed the questionnaire package. Based on their MAAS scores, we identified 31 participants as high mindfulness (top 25% of all the participants) and 31 participants as low mindfulness (bottom 25% of all the participants). Later, 23 participants in the high mindfulness group and 26 in the low mindfulness group agreed to participate in the experiment (*M* age = 18.96 yrs, SD = 1.08; females = 23, males = 26). Before participating in the formal experiment, those 49 participants signed a consent form and participated in the formal experiment. We used G ∗Power to estimate the experiment sample size. With a 2 by 2 mixed design and set alpha = 0.05, the estimated sample size was 48. Descriptive data for participants are presented in Table 1.

**Table 1:** Demographic characteristics of participants (*N* = 49).

Measures	Male	Female	High mindful group	Low mindful group	Total
*n*	26	23	26	23	49
Age	19.08 ± 1.32	18.83 ± 0.72	18.69 ± 0.62	19.26 ± 1.39	18.96 ± 1.08
Height	174.58 ± 6.00	160.78 ± 5.41	168.69 ± 10.	167.43 ± 6.5	168.10 ± 8.96
Weight	69.31 ± 14.20	51.43 ± 6.76	61.62 ± 17.0	60.13 ± 11.0	60.92 ± 14.39
BMI	22.78 ± 4.74	19.87 ± 2.15	21.38 ± 4.26	21.44 ± 3.77	21.41 ± 4.00

**Notes.**

BMI, body mass index.

### Phase 2: formal experiments

#### Nature of self-talk

To allow participants to learn self-talk skills, we developed a self-talk script with a professor of sports psychology and an expert familiar with psychological skill training. Then, we displayed this self-talk script on an instruction sheet before performing the assigned motor task. Two types of self-talk (i.e., instructional and unrelated self-talk) were applied to perform two motor tasks: standing long jump and a line tracking test. To apply instructional self-talk for the line tracking test, participants had to talk to themselves the words like “focus on the center of the groove of the panel and move it as fast as possible!” To apply unrelated self-talk for the line tracking test, we refer to Chang et al.’s (2014, p.141) method by asking participants to talk to themselves with words like “ the weather today, my clothes’ colors, or my pets’ names.” To apply instructional self-talk for the standing long jump, participants had to talk to themselves with words like “look at the front landing spot, swing the arms, relax, and jump forward as far as possible!” To apply unrelated self-talk for the standing long jump, participants had to talk to themselves with words like “ the weather today, my clothes’ colors, or my pets’ names.”

#### Self-talk manipulation check

We adopted Kolovelonis and colleagues’ suggestion (2011) to check whether participants followed our design in performing motor tasks under different self-talk interventions. We used a self-talk check question for participants after each performance attempt. The question was “during standing long jump/line tracking tasks did you use the self-talk from self-talk sheet as is instructed?” The response options ranged from “ *1* ” absolutely not to “*5*” completely so.

#### Motor performance measure

##### Standing long jump task.

We adopted Westphal and Porter’s (2013) suggestion by using the standing long jump to test participants’ discrete motor skill performance. The standing long jump was used to assess participants’ muscular power and strength. The task was conducted in a regular standard-sized plastic pad with two lines indicating the distance in centimeters. During the test, the participants were instructed to perform warm-up until ready to act (about 3–4 min). To begin with the experiment, participants had two warm-up attempts with 30% and 60% intensity of jumping. Then, the participants were asked to execute six maximal attempts for official testing and asked to jump as far as possible. The longest distance was recorded for further analysis.

#### Hand-eye coordination of fine line tracking test

We used the Austrian made “Visual Pursuit Test of the Vienna Test System (VTS, Schuhfried GmbH) for the experiment. The VTS is a computerized psychological assessment tool for perceptual-motor skill testing (Schmid et al., 2005). We used Motor Performance Series Workboard (MLS), one of the devices of VTS to measure participants’ continuous motor skill performance. According to Fleishman’s (1954) conceptualization of perceptual-motor skill tests, MLS is a very reliable and valid test for continuous motor skills. The MLS requires dynamic and static ability in coordinating finger-hand-arm movement. There is a path-way in the MLS task with several geometric figures that requires participants to perform a single hand line tracking. The size of the work board is 300 × 300 ×  15 mm, and there are several holes, grooves, and contact surfaces. There are two pens on the work board, one is black on the right and one is red on the left. In this study, participants used their preferred hand in sitting position to perform the motor task, but their bodies cannot touch any part of the work board. To begin with the test, participants pick up the electronic pen and touch the starting point, then move along with the path of the groove until touching the finish point. While performing the task, the electronic pen cannot leave the upper edge of the work board nor touch the side or bottom. If the pen hits the edge of the groove, it is recorded as a mistake. More errors represent poorer performance in the line tracking motor skill task. We used frequencies of mistakes for further analysis.

### Experimental procedures

Prior to data collection, we gained approval from the local institutional review board (TSMH IRB No.18/Protocol No.108-B). Before formal experiments, all participants were given a short lecture to introduce the purpose of our study, the content of self-talk, and the procedures to perform motor tasks. To avoid Hawthorne effects (Thomas, Nelson & Silverman, 2015), we just told the participants that this is an experiment to examine the relationship between language use and motor performance. What they need to do was just try their best during the experiment. For formal experiments, participants experienced two self-talk conditions with counterbalanced order. The participants were asked to select the self-talk condition by drawing lots. Then participants completed six trials of standing long jump and four trials of line tracking test. To avoid fatigue effects, we gave participants a 5-minute break after standing long jump. Also, during each trial of line tracking, we gave 3 min of break. As stated earlier, the longest standing long jump and the best line tracking performance (i.e., the fewest mistakes) were used for further analysis. Before they performed the official attempt, the experimenters displayed the self-talk scripts. Participants selected one self-talk item from the script and then verbally and loudly spoke the script to make sure they understand the words. After this step, the participants started to perform a formal test. Before each trial, they had to speak these self-talk scripts either loudly or silently before performing the motor tasks. The self-talk manipulation check was then administrated to confirm the appropriate use of specific self-talk following each official attempt. All these experiments were conducted in a quiet room of a sport psychology lab. When experiments were conducted, no other person was present in order to reduce social facilitation effects. Further, to avoid other unnecessary effects on experiments, we did not provide any feedback on the performance. It took about 40 min for each participant to complete the whole experiment.

### Statistical analyses

Two separate 2 × 2 (self-talk × high vs. low mindfulness disposition) mixed design ANOVA were used to compare the effects of self-talk and high vs. low mindfulness disposition on standing long jump and line tracking task. Significant effects were followed-up with multiple comparisons with Bonferroni correction and Greenhouse Geisser correction in order to meet the sphericity assumption. The significance level was set at alpha level of .05 prior to Bonferroni correction.

## Results

Regarding the self-talk manipulation check, participants were able to use the self-talk requested for the specific condition during both motor tasks (*M* = 4.88∼4.94, SD = 0.24∼0.33). Because the highest possible score is 5, the scores indicated that participants followed the experimental design to use different types of self-talk. The descriptive data on motor task performance as well as a mindfulness group for the two self-talk conditions are presented in Table 2.

**Table 2:** Descriptive statistics for all participants on two motor tasks under different self-talks.

Variable	Instructional	Unrelated
Standing Long Jump (*N* = 49)	171.82 ± 40.21	166.88 ± 37.15
Low mindfulness (*n* = 23)	171.17 ± 38.20	166.30 ± 35.07
High mindfulness (*n* = 26)	172.38 ± 42.65	167.38 ± 39.59
Line Tracking Test (*N* = 49)	13.18 ± 7.16	16.36 ± 10.31
Low mindfulness (*n* = 23)	14.39 ± 6.95	19.83 ± 12.05
High mindfulness (*n* = 26)	12.12 ± 7.31	13.29 ± 7.47

### Interaction effects of mindfulness and self-talk in standing long jump

The 2 by 2 mixed design ANOVA showed no significant self-talk × mindfulness group interaction in standing long jump, *F* (1,47) = 0.001, *p* = .97. However, the analysis revealed a main effect of self-talk on standing long jump, *F* (1, 47) = 6.22, *p* = .02, *η*^2^ = .12, indicating that participants performed better under instructional self-talk than unrelated self-talk. Further, there was no main effect of mindfulness group for standing long jump, *F* (1,47) = 0.01, *p* = .92.

### Interaction effects of mindfulness and self-talk in line tracking

**Figure 1:**
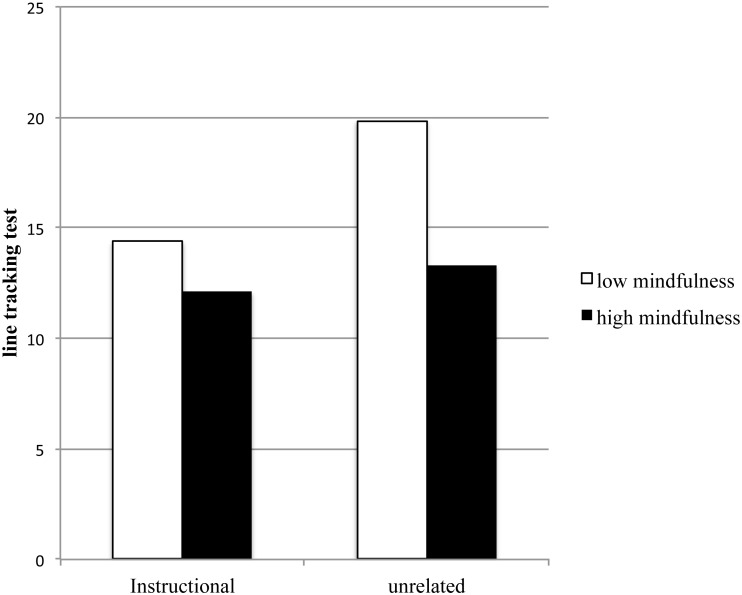
Interaction of mindfulness and two types of self-talk on line tracking. This figure illustrates how high vs. low mindfulness disposition performed differently on line tracking under different self-talks.

Regarding line tracking, the 2 by 2 mixed design ANOVA showed a significant self-talk × mindfulness group interaction, *F* (1, 47) = 6.12, *p* = .02, *η*^2^ = .12 (see Fig. 1). Further, we utilized both independent *t*-test and paired *t*-test to compare this simple main effect of line tracking between mindfulness and self-talk. Under instructional self-talk, there was no difference between high vs. low mindfulness participants in line tracking performance. However, under unrelated self-talk, the independent *t*-test showed that high mindfulness performed better than low mindfulness in line tracking, *t* (47) = 2.31, *p* = .01, Cohen’s *d* = 0.66. Further, the paired *t*-test showed that low mindfulness participants performed better under instructional self-talk than unrelated self-talk, *t* (22) = 3.38, *p*= .00, Cohen’s *d* = 0.70. However, for high mindfulness participants, there was no difference in line tracking between instructional self-talk and unrelated self-talk.

## Discussion

### Theoretical Implications

In considering that mindfulness disposition might moderate the effects of self-talk on motor task performance, the present study recruited participants with high vs. low mindfulness to perform standing long jump and line tracking under instructional self-talk and unrelated self-talk conditions. Results found several novel findings. First, an interaction effect was found between mindfulness and self-talk in line tracking. Specifically, low mindfulness participants performed poorer than high mindfulness participants in line tracking task under unrelated self-talk. Further two main effects indicated that participants performed better in standing long jump and line tracking under instructional self-talk. However, high vs. low mindfulness participants did not differ in standing long jump and line tracking. The results provide several implications for researchers.

First, our results confirmed that self-talk is beneficial to motor performance (e.g., Dickens, Van Raalte & Hurlburt, 2018; Hardy, Comoutos & Hatzigeorgiadis, 2018; Weinberg, Miller & Horn, 2012). Specifically, we found that all participants performed better both in standing long jump and line tracking under the instructional self-talk condition. The effects of instructional self-talk on motor performance may be due to performers focusing on relevant cues in the environment and specific technical/mechanical aspects of the skills compared to those who did not receive instructions (Abdoli et al., 2018; Boroujeni & Shahbazi, 2011; Chang et al., 2014; Theodorakis et al., 2000).

The interaction of mindfulness disposition and self-talk in line tracking is very insightful for researchers. To the best of our knowledge, this is the first study that includes mindfulness disposition in examining the effects of self-talk on motor performance. Although past researchers (e.g., Theodorakis et al., 2000; Zourbanos et al., 2013) suggested that attention might explain the mixed results of the effects of the instructional self-talk on motor performance, no study has examined the role of mindfulness disposition in self-talk-motor performance relationship. Our study used mindfulness disposition as a moderator, and indirectly supported the moderating role of attention between instructional self-talk and motor performance relationship. Future study may use a direct measure of attention, such as EEG and other neuro-physiological index, to examine the moderation effect of attention on the self-talk- motor performance relationship (e.g., Martel, Dahne & Blankertz, 2014).

The interaction effect of mindfulness and self-talk on continuous motor task also outlines the importance of instructional self-talk for low mindfulness participants. Especially, we found low mindfulness participants performed better in line tracking only under instructional self-talk. The results can be explained that instructional self-talk providing task-relevant cues or information so they can perform well even with low ability to sustain their attention (Hardy, 2006; Hardy, Gammage & Hall, 2001; Hatzigeorgiadis, Theodorakis & Zourbanos, 2004). However, the results also found those participants with high mindfulness disposition did not differ under instructional self-talk or unrelated self-talk in two motor tasks. This can be explained in that their high ability to sustain attention from moment to moment helped them perform better with different tasks either under instructional or unrelated self-talk conditions. Kee & Liu (2011) suggested that high mindfulness participants performed better in different motor tasks because they are kinesthetically aware and become responsive to motor tasks required. Thus, even under unrelated self-talk condition, their performance was not impaired.

Further, under unrelated self-talk, high vs. low mindfulness participants had no difference in standing long jump but had a significant difference in line tracking is worthy of discussion. This unique result can be explained in that a discrete motor task such as standing long jump, is a rapid and ballistic movement that requires a generalized motor program (GMP Schmidt, 1975) to perform the task. It requires a short time to sustain attention so it does not interfere low mindfulness participants’ performance under unrelated self-talk. In contrast, a continuous motor task such as line tracking requires performers to sustain their attention to accomplish the task. If during a continuous task a competing attention appeared (e.g., unrelated self-talk), it would impair participants’ performance, especially for those with low mindfulness disposition (Schmidt & Lee, 2011, pp. 103–105). The unrelated self-talk can be a source of distraction for line tracking that distracts low mindfulness performers’ attention and makes errors. The central-resource capacity theories of attention (McGill, 2004; pp. 143–145) proposed that there is motor performance deficit when performing multiple activities at the same time for novel tasks or unexperienced performers. Because there is limited capacity to receive and handle information, if performers are beginners or unexperienced, they have difficulty receiving correct information to react correctly. For our study, because low mindfulness participants tend to be less able to sustain their attention, talking to themselves with unrelated words would distract their attention during line tracking. In contrast, high mindfulness participants are characterized by an ability to maintain attention from moment to moment. They were less distracted when speaking unrelated words. Therefore, further studies are needed to examine the performers’ mindfulness disposition, the role of attention allocation, type of self-talk, and motor task.

### Limitations and future suggestions

Despite these significant and unique findings, several limitations should be considered when interpreting the results of the present study. First, the operational and conceptual equating of mindfulness disposition and attention ability may be a limitation, although related literature suggests that high mindfulness disposition indicates high attention (Brown & Ryan, 2003). Also, empirical research supports that mindfulness induction can enhance attention through the evidence of left dorsomedial prefrontal cortex activities (Doll et al., 2016), but we did not measure left dorsomedial prefrontal cortex activities. Therefore, whether attention moderates the effects of instructional self-talk on motor performance needs to be further examined. We suggest that future studies may examine the effects of instructional self-talk on motor performance and simultaneously measure EEG activities. By so doing, the role of attention in the instructional self-talk-motor performance relationship can be further understood.

In addition, our study was conducted in a laboratory setting where participants were asked to perform motor tasks under different self-talk conditions. Whether the results are applicable to real athletic settings needs to be further examined. Specifically, a recent meta-analysis found male expert athletes performed better on cognitive-motor tasks which require attentional abilities (Voss et al., 2010). Thus, future research needs to examine the effects of instructional self-talk on motor tasks when participants are expert athletes or have sporting experience.

Further, because the duration of our self-talk intervention in the present study was adopted from previous research (e.g., Chang et al., 2014; Boroujeni & Shahbazi, 2011; Theodorakis et al., 2000), whether a long-term intervention produces similar results needs to be further examined. Specifically, some studies have conducted relatively long-term self-talk programs (4 to 10 weeks) and support their effectiveness for sports performance (Hamilton, Scott & MacDougall, 2007; Hatzigeorgiadis et al., 2014; Zetou et al., 2012). Moreover, we suggest future studies examine the effects of instructional self-talk in varied settings. Martin, Vause & Schwartzman (2005) indicated that most self-talk research is conducted in laboratory settings. Therefore, future studies may examine how instructional self-talk in competitive sports settings, academic settings, fitness centers, military, sports injury, and rehabilitation. Moreover, we found no difference in discrete and continuous tasks for high vs. low mindfulness participants. The true reasons for this finding are unknown. We suggest future studies to examine how mindfulness disposition interacts with external stimuli on different motor tasks under different self-talk conditions.

### Applications

Practitioners, such as PE teachers, coaches, and sport psychology consultants can apply our results in various settings. To facilitate students or athletes’ motor learning and control practitioners should provide positive and task-relevant information to performers (Edwards, 2011; pp. 362–401). Specifically, practitioners should provide both the general and specific aspects of the skills so performers can understand the key components of the skills and direct their attention to those parts when performing the motor skills. Also, practitioners should avoid giving unrelated information to prevent distracting attention. In addition, because there is limited information processing ability for beginners or unexperienced students, it is suggested that PE teachers, coaches, and sports psychology consultants should give each key instruction each time when learning a new skill. Also, the instructors should give precise information about the skill requirements and avoid jargon (Edwards, 2011, pp. 366-367). For those students with high mindfulness, it is suggested that PE teachers and coaches could provide a different level of assignments and materials, and foster independent learning (Laine & Tirri, 2015).

## Conclusion

Self-talk is a very useful psychological skill to enhance performers’ motor skill learning and control. Our study provides evidence that instructional self-talk is beneficial to both discrete and continuous motor skills. Additionally, we found participants’ mindfulness disposition interacts with self-talk on the motor task. Future research may extend our research by directly examining performers’ mindfulness disposition, the role of attention allocation, type of self-talk, and motor task to unveil the interweaving relationships of instructional self-talk, attention, and motor performance. Moreover, when applying instructional self-talk with athletes and PE students, practitioners should provide positive and task-related instruction in order to facilitate performers’ motor task performance.

##  Supplemental Information

10.7717/peerj.7034/supp-1 Supplemental Information 1 Raw data for the interaction of mindfulness and self-talk This table illustrates all the statistics of all participants’ performance on two types of motor tasks under different self-talk.

## Additional Information and Declarations

### Data Availability

The following information was supplied regarding data availability: Raw data are available as Supplemental File.
